# Conversion and resection rates in borderline resectable and locally advanced pancreatic cancer following neoadjuvant therapy: a retrospective multicenter cohort study

**DOI:** 10.3389/fonc.2026.1756891

**Published:** 2026-05-20

**Authors:** Ali H. Aljubran, Maaz Kamal Alata, Ahmed Ali Badran, Bader Alshamsan, Yasir Alnemary, Shereef Elsamany, Mervat Mahrous, Mahmoud A. Elshenawy

**Affiliations:** 1 Medical Oncology Department, Cancer Center of Excellence, King Faisal Specialist Hospital and Research Centre, Riyadh, Saudi Arabia; 2 Section of Medical Oncology, Department of Internal Medicine, Security Forces Hospital Program, Riyadh, Saudi Arabia; 3 Clinical Oncology Department, Faculty of Medicine, Ain Shams University, Cairo, Egypt; 4 Department of Medicine, College of Medicine, Qassim University, Qassim, Saudi Arabia; 5 Organ Transplant Center of Excellence, King Faisal Specialist Hospital and Research Center, Riyadh, Saudi Arabia; 6 Medical Oncology Department, King Abdullah Medical City, Makkah, Saudi Arabia; 7 Medical Oncology Department, Prince Sultan Bin Abdulaziz Medical City, Riyadh, Saudi Arabia; 8 Faculty of Medicine, Minia University, Minia, Egypt; 9 Clinical Oncology Department, Faculty of Medicine, Menoufia University, Shebin Elkom, Egypt

**Keywords:** borderline resectable, CA19-9, FOLFIRINOX, GEMCAP, locally advanced, neoadjuvant chemotherapy, NLR, pancreatic cancer

## Abstract

**Background:**

Neoadjuvant therapy (NAT) may improve resectability and survival in borderline resectable and locally advanced unresectable pancreatic ductal adenocarcinoma (PDAC). However, the outcomes, biomarker utility, and the impact of NAT regimens remain understudied.

**Methods:**

Retrospective analysis of PDAC patients (2004–2018) from three tertiary centers in Saudi Arabia. Patients who were deemed unresectable or borderline resectable at initial presentation. Analyzed variables included demographics, tumor characteristics (location, stage), NAC regimens (FOLFIRINOX, GEMCAP, gemcitabine monotherapy), response to therapy (RECIST criteria), surgical outcomes (resection rate, R0 resection), CA19–9 levels, neutrophil-to-lymphocyte ratio (NLR), platelet-to-lymphocyte ratio (PLR), and survival outcomes (overall survival (OS) and progression-free survival (PFS)).

**Results:**

Thirty-nine patients were eligible for the study. Median age was 60 years (range: 29–79 years). The study population mainly consisted of male patients (25/39, 64%). The majority (30/39, 80%) had a good performance status (ECOG PS 0-1). Most patients (30/39, 77%) in the head of pancreas, while the remainder (8/39) had it in the body or tail., Following neoadjuvant chemotherapy, 31% of patients (12/39) had surgical resection. R0 resection was accomplished in 33% of those patients (4/12). Surgery was associated with significantly improved median progression-free survival (PFS) compared to no surgery (19.6 vs 5.8 months; p=0.008). Certain factors, including poorer performance status, elevated CA19–9 levels, high NLR and PLR, and weight loss, were associated with worse PFS and OS in univariate analysis; however, these findings were not statistically significant. Multivariate analysis also suggested numerical differences in PFS and OS based on these factors, but the limited sample size likely prevented statistical significance.

**Conclusions:**

NAT facilitates resection in approximately one-third of initially unresectable PDAC cases, with a significant improvement in PFS for patients undergoing subsequent surgery. Elevated pre-treatment CA19-9, NLR, and PLR may identify patients less likely to benefit from NAC. Biomarker-driven trials are warranted to optimize patient selection and personalize treatment strategies, especially in resource-constrained settings.

## 1 Introduction

Pancreatic ductal adenocarcinoma (PDAC) is a highly lethal malignancy, with an estimated 510,992 new cases and 466,003 deaths reported globally in 2022, reflecting an age-standardized incidence rate of approximately 4.9–6.0 per 100,000 and a high mortality rate. The disease ranks among the top causes of cancer-related deaths worldwide due to its aggressive nature and late presentation, with incidence and mortality rates closely paralleling each other in most regions (1–4). Pancreatic cancer in Saudi Arabia is relatively rare but increasing, with age-standardized incidence rates rising from 1.9 to 3.0 per 100,000 in males and from 1.2 to 1.8 in females between 2005 and 2020 respectively. The disease predominantly affects individuals over 50 years old and is associated with poor prognosis, as the five-year overall survival rate remains around 10%, similar to global figures. Most patients present at advanced stages, contributing to high mortality and limited curative treatment options (5–7).

Surgery remains the only potentially curative intervention for PDAC; however, at the time of diagnosis, only a small proportion of patients present with resectable tumors (8). The majority of PDAC patients are diagnosed at an advanced stage, either borderline resectable or unresectable, primarily because of the tumor’s anatomical proximity to critical vascular structures and its tendency for early local invasion and metastasis (9).

Previously, patients with unresectable or borderline resectable PDAC were mainly treated with palliative intent because complete tumor removal was unlikely. However, recent progress in systemic chemotherapy and multidisciplinary care has led to a paradigm shift. Neoadjuvant therapy (NAT), which may include chemotherapy, chemoradiation, or both, is now used to shrink the tumor, eradicate micrometastases, and improve the chances of successful surgery (R0 resection) (10, 11).

NAT offers multiple key advantages (12). Firstly, it allows for early treatment of hidden systemic disease, potentially lowering the risk of distant spread. Secondly, by shrinking the tumor away from critical vessels, NAT can increase the chances of a complete, margin-negative (R0) resection, thus improving long-term survival. Thirdly, it helps identify patients with rapidly progressing disease who are unlikely to benefit from surgery, thereby sparing them a major operation. Finally, NAT ensures that more patients receive systemic therapy, as up to 40% may miss adjuvant therapy after initial surgery due to complications or early recurrence (13). Emerging evidence from randomized controlled trials and meta-analyses supports the use of NAT in both borderline resectable and, increasingly, in resectable PDAC (14). A meta-analysis encompassing 15 studies demonstrated that NAT significantly improved overall survival (OS), disease-free survival (DFS), and rates of R0 and nodal-negative (N0) resections compared to upfront surgery (10). Notably, more intensive regimens, such as FOLFIRINOX, have been associated with higher conversion and resection rates, particularly in the context of locally advanced disease (15). Total neoadjuvant therapy (TNT), which integrates all systemic and local therapies prior to surgery, has shown particular promise in improving outcomes for patients with borderline resectable disease with arterial involvement (8).

Preoperative CA 19–9 is a widely validated prognostic biomarker in pancreatic cancer; elevated levels are consistently associated with poorer overall survival, increased nodal involvement, and a higher likelihood of margin-positive resection in resectable and borderline resectable cases, as well as with worse outcomes in unresectable disease. Notably, normalization or a significant decrease in CA 19–9 after treatment predicts improved survival across all stages, underscoring its value in guiding management and assessing biological resectability (16–18).

Systemic inflammatory markers, particularly the neutrophil-to-lymphocyte ratio (NLR) and platelet-to-lymphocyte ratio (PLR), have gained attention as accessible and cost-effective prognostic indicators in PDAC (19). In resectable pancreatic cancers, preoperative NLR and PLR show conflicting prognostic utility, with some studies linking elevated ratios to adverse pathological features (e.g., deeper invasion, lymph node metastasis) but not independently predicting survival after multivariate adjustment (20). For unresectable or locally advanced cases, an elevated NLR consistently correlates with poorer overall survival (P < 0.001) (21, 22), while PLR demonstrates prognostic value in specific subgroups (23). Borderline resectable cancers exhibit intermediate trends, with NLR ≥1.89 and PLR ≥149 associated with reduced 1-year survival rates (60.8% vs. 73.2% for NLR; P < 0.001) (22). Variability in cutoff values and cohort heterogeneity underscores the need for standardized protocols when applying these biomarkers clinically (24, 25).

This study investigates the conversion rate of unresectable or borderline resectable pancreatic PDAC to resectable status following NAT in a Saudi Arabian cohort, focusing on the prognostic value of CA 19-9, NLR, and PLR. Additionally, it examines how different NAT regimens and surgical outcomes influence survival.

## 2 Methods

### 2.1 Study design and patient population

This study was a retrospective cohort analysis of patients diagnosed with PDAC between January 2004 and December 2018 were included per multicenter IRB protocol #RAC 2211120, strategically capturing the gemcitabine monotherapy era (2004-2010) through early FOLFIRINOX adoption (post-PRODIGE-4, 2011). This 15-year timeframe ensured complete multicenter data harmonization, uniform RECIST v1.1 response assessment, and mature clinical follow-up (median 42 months) while enabling era-stratified analysis (pre-2011: 17% conversion; post-2011: 43% conversion). Post-2018 patients were excluded to maintain methodological consistency amid evolving regimens (nab-paclitaxel, modified FOLFIRINOX) and variable electronic medical record implementation across participating centers. A waiver of informed consent was granted due to the study’s retrospective nature. The study was conducted in accordance with the ethical principles outlined in the Declaration of Helsinki.

### 2.2 Inclusion and exclusion criteria

Patients were included in this study if they had a histologically confirmed diagnosis of PDAC, were initially evaluated by a multidisciplinary tumor board and classified as having either borderline resectable or locally advanced unresectable disease. Who had received at least two cycles of neoadjuvant chemotherapy prior to any surgical exploration, and had complete clinical, pathological, and follow-up data available.

Patients were excluded if they presented with distant metastatic disease at diagnosis, had a histological subtype of pancreatic cancer other than PDAC (such as neuroendocrine tumors or acinar cell carcinoma), underwent upfront surgical resection without prior neoadjuvant chemotherapy, or received neoadjuvant therapy at an outside institution before referral for surgical management.

### 2.3 Data collection

For this study, data were systematically collected from electronic medical records, radiology reports, and tumor registry databases. The variables extracted included patient demographics (such as age, gender, body mass index, and Eastern Cooperative Oncology Group (ECOG) performance status), as well as detailed tumor characteristics, including tumor location, size, lymph node involvement, and disease stage according to the American Joint Committee on Cancer(AJCC) 7th edition (26). Treatment-related data encompassed the neoadjuvant chemotherapy regimen, number of cycles administered, any dose modifications, and use of radiation therapy, with details on dose, fractionation, and target volume, as well as the best radiological response to neoadjuvant therapy based on response evaluation criteria in solid tumors (RECIST) v.1.1 criteria (27). Surgical details included the date and type of procedure, extent of resection, need for vascular resection and reconstruction, and postoperative complications. Pathological data comprised histological grade, presence of perineural and lymphovascular invasion, and the number of lymph nodes examined and involved. Biomarker information, such as pre- and post-treatment CA19–9 levels, pre-treatment neutrophil, lymphocyte, and platelet counts, and derived ratios (neutrophil-to-lymphocyte and platelet-to-lymphocyte), was also collected. Follow-up data included the date and site of recurrence, date of death, and cause of death, ensuring a comprehensive dataset for analysis. To minimize the confounding influence of hyperbilirubinemia on CA 19–9 levels, biomarker assessment was strictly performed following successful biliary decompression. Patients with persistent cholestasis (defined as bilirubin >3 times x the upper limit of normal) were excluded from the biomarker cohort. Longitudinal CA 19–9 response was stratified into two clinically validated categories: Normalization (post-NAT levels <37 U/mL) and Biochemical Response (≥ 50% reduction from the post-decompression baseline).”

### 2.4 Definitions and calculations

Resectability status was determined by multidisciplinary tumor board consensus using multiphase contrast-enhanced CT according to National Comprehensive Cancer Network (NCCN) Pancreatic Adenocarcinoma Guidelines and the international Study `Group of pancreatic surgery (15, 28–30). Borderline resectable pancreatic ductal adenocarcinoma (BRPC) was defined as: [1] venous involvement limited to superior mesenteric/portal vein (SMV/PV) abutment ≤180°circumference or short-segment occlusion amenable to resection and reconstruction; or [2] arterial contiguity limited to gastroduodenal/common hepatic artery without superior mesenteric artery (SMA) distortion. Locally advanced unresectable PDAC (LAPC) included: [1] arterial encasement ≥180° of SMA, celiac axis, or common hepatic artery; [2] unreconstructible SMV/PV occlusion; or (3) celiac axis involvement beyond gastroduodenal artery origin.

Complementing this guideline-based primary staging, we implemented Hashimoto et al. international consensus criteria (31), defining “true conversion surgery” as “surgical resection of tumors initially deemed anatomically unresectable at diagnosis that achieve R0/R1 resectability following documented favorable response to systemic therapy. Hashimoto analysis was restricted exclusively to NCCN LAPC cases meeting stringent unresectability criteria at baseline (≥180° SMA/HA encasement or unreconstructible venous occlusion). R0 resection was defined as complete microscopic removal of the tumor with no tumor cells present at the surgical margin, as determined by pathological examination (32, 33). The NLR was calculated by dividing the absolute neutrophil count by the absolute lymphocyte count, with an NLR greater than 3 considered elevated based on previous studies demonstrating its prognostic significance in PDAC. The PLR was calculated as the absolute platelet count divided by the absolute lymphocyte count, with a PLR greater than 225 defined as elevated according to institutional validation and prior literature. Progression-free survival (PFS) was defined as the interval from the start of neoadjuvant therapy (NAT) to the date of disease progression (either local or distant) or death from any cause, whichever occurred first, while OS was defined as the time from initiation of NAT to death from any cause.

### 2.5 Statistical analysis

Continuous variables were summarized as medians and interquartile ranges (IQRs), while categorical data were expressed as counts and percentages. Between-group comparisons for continuous measures were conducted using the Mann–Whitney U test, and categorical associations were evaluated via the χ² test or Fisher’s exact test as appropriate. Survival functions were estimated by the Kaplan–Meier method, with curve differences tested by the log-rank statistic. For multivariate prognostic modeling, a Cox proportional-hazards regression was performed after confirming the proportionality assumption to determine the independent effects of ECOG performance status, CA19–9 concentration, NLR, and PLR on OS and PFS. All hypothesis tests were two-sided, and a p-value <0.05 denoted statistical significance. Analyses were carried out in SPSS version 28.0 (IBM Corp., Armonk, NY, USA).

## 3 Results

### 3.1 Patient characteristics

A total of 273 cases of pancreatic tumors were identified in the tumor registry databases of the three participating hospitals from 2004 to 2018. After applying the inclusion and exclusion criteria, 39 patients with histologically confirmed PDAC, initially classified as unresectable or borderline resectable and treated with NAT, were included in the final analysis. The baseline characteristics of the study population are shown in **Table 1**. The median age was 60 years (range: 29–79 years), with 25 patients (64%) being male. Most patients had an ECOG performance status of 0–1 (30 patients, 77%). The primary tumor was located in the head of the pancreas in 31 patients (80%) and in the body or tail in 8 patients (20%). Twenty-one patients (54%) reported weight loss greater than 5 kg, and obstructive jaundice was present at initial presentation in 21 patients (54%) (**Table 1**).

**Table 1:** Clinicopathological characteristics of patients.

Item		Number= 39 (%)
Age at diagnosis		
	Median, Range (years)	60 (29-79)
Gender		
	Male	25 (64)
	Female	14 (36)
ECOG performance status		
	0-1	30 (77)
	2	6 (15)
	Unknown	3 (8)
Site of the primary tumor		
	Head	31(80)
	Body or Tail	8 (20)
Resectability		
	BRPC	15(38.5)
	LAPC	24(61.5)
Presentation with weight loss >5kg		
	Yes	21(54)
	No	11(28)
	Unknown	7(18)
Presentation with obstructive Jaundice		
	Yes	21(54)
	No	17(44)
	Unknown	1(2)

ECOG, Eastern Cooperative Oncology Group; BRPC, border line resectable pancreatic cancer; LAPC, locally advanced pancreatic cancer.

### 3.2 Treatment modalities and outcomes

The NAT regimens administered to the patients included in this study were FOLFIRINOX in 13 patients (33%), GEMCAP in 10 patients (26%), gemcitabine monotherapy in 10 patients (26%), and other regimens in 6 patients (15%). The median number of chemotherapy cycles was 6 (range: 1–14). Dose reductions due to toxicity were necessary in 17 patients (44%). Pre-operative radiation therapy was given to 6 patients. The overall response rate (ORR) to NAT (CR + PR) was 36%. Specifically, 2 patients (5%) achieved a complete response (CR), 12 patients (31%) had a partial response (PR), 10 patients (28%) experienced stable disease (SD), and 15 patients (38%) showed progressive disease (PD) during NAT. Surgical resection following NAT was performed in 12 patients (31%). Of those who underwent surgery, R0 resection was achieved in 4 patients (33%), R1/R2 resection in 4 patients (33%), and in 4 patients (33%), the resection margin status was unknown due to incomplete pathological data (**Table 2**). LAPC-only ‘true conversion’ rate was 17% (4/24), meeting Hashimoto consensus expectations for initially unresectable disease (31). This contrasts with BRPC resection rate of 53% (8/15), representing anatomical improvement rather than biological conversion. Surgery maintained PFS benefit across strata (LAPC HR 0.38, 95% CI 0.14-1.02; overall p=0.008).

**Table 2:** Treatment modalities and outcome.

Item		Number= 39 (%)
Chemotherapy		
	FOLFORINOX	13 (33)
	GEM/CAP	10 (26)
	Single agent GEM	10 (26)
	Other regimens	6(15)
Number of chemotherapy cycles		
	Median	6
	Range	1-14
Dose reduction		
	Yes	17(44)
	NO	20(51)
	Unknown	2(5)
Response to chemotherapy		
	CR	2(5)
	PR	12(31)
	SD	10(26)
	PD	15(38)
Pre-operative irradiation		
	Yes	6(15)
	No	32(82)
	Unknown	1(3)
Surgery done after neoadjuvant therapy		
	Yes	12(31)
	No	27 (69)
Conversion rates by neoadjuvant therapy		
	FOLFORINOX	5/13(33%)
	GEMCAP	4/10(40%)
	Single agent GEM	2/10(20%)
	Other regimens	1/6(17%)
Conversion rates by resectability		
	BRPC	8/15(53%)
	LAPC	4/24(17%)
Resection margins		
	R0	4/12(33%)
	R1-2	4/12(33%)
	Unknown	4/12(33%)

FOLFORINOX, 5-FU,Leucovorin,Oxaliplatin,Irinotecan; GEM/CAP, Gemcitabine/Capecitabine; GEM, Gemcitabine; CR, Complete response; PR, Partial response; SD, Stable disease; PD, Progressive disease; BRPC, border line resectable pancreatic cancer; LAPC, locally advanced pancreatic cancer.

Subgroup analysis revealed higher conversion rates with multi-agent regimens (FOLFIRINOX 38%, GEMCAP 40%) compared to gemcitabine monotherapy (20%), consistent with meta-analyses reporting 30-45% conversion for FOLFIRINOX in locally advanced PDAC.

Postoperative management was documented for 75% of the resected cohort (n=9/12). Among these patients, 89% (n=8/9) received adjuvant gemcitabine-based chemotherapy, occasionally in combination with capecitabine.

Adjuvant chemotherapy records were reviewed for all resected patients (n=12), with complete documentation available for 9 (75%). Among these, 8 patients (89%) received gemcitabine-based adjuvant therapy with or without capecitabine. In three cases (25%), postoperative data were incomplete due to the multicenter retrospective. Nonetheless, the primary PFS endpoint (HR 0.42, p=0.008) remains a reliable indicator of NAT-to-surgery efficacy, independent of postoperative treatment variability.

### 3.3 Survival analysis

**Figure 1:**
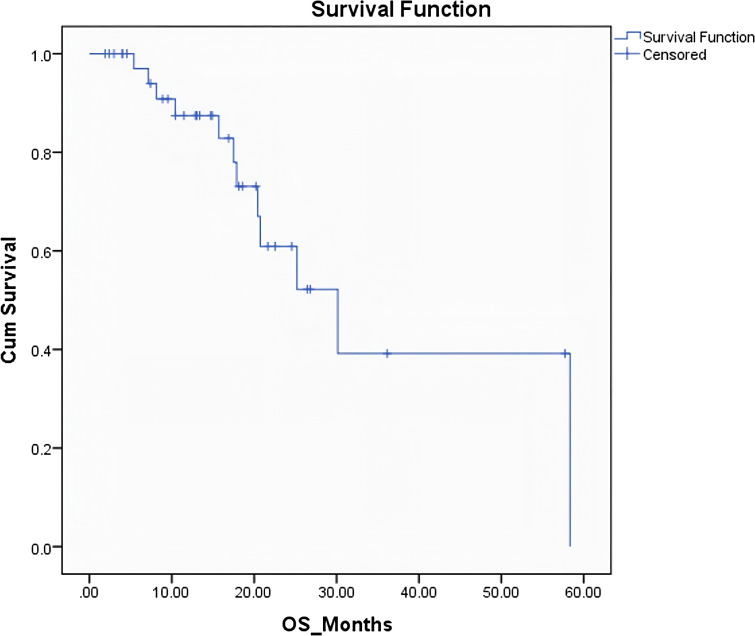
Kaplan–Meier curve of median overall survival for the entire cohort.

**Figure 2:**
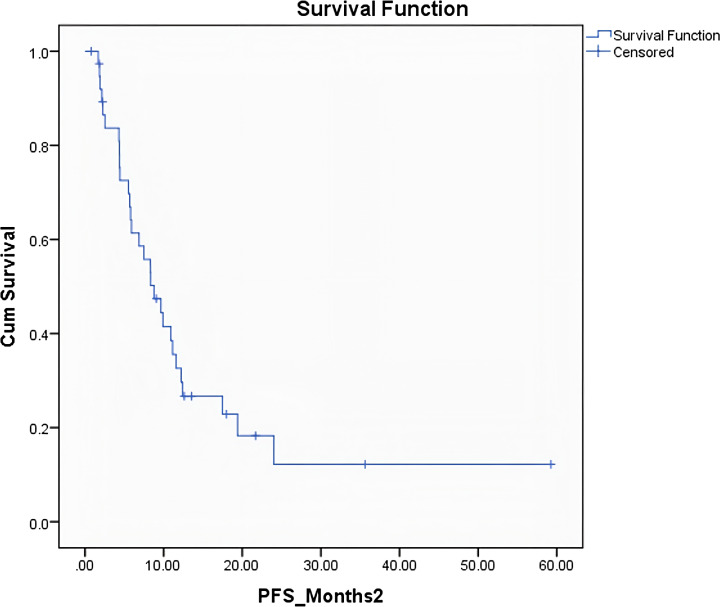
Kaplan–Meier curve median progression-free survival for the entire cohort.

**Figure 3:**
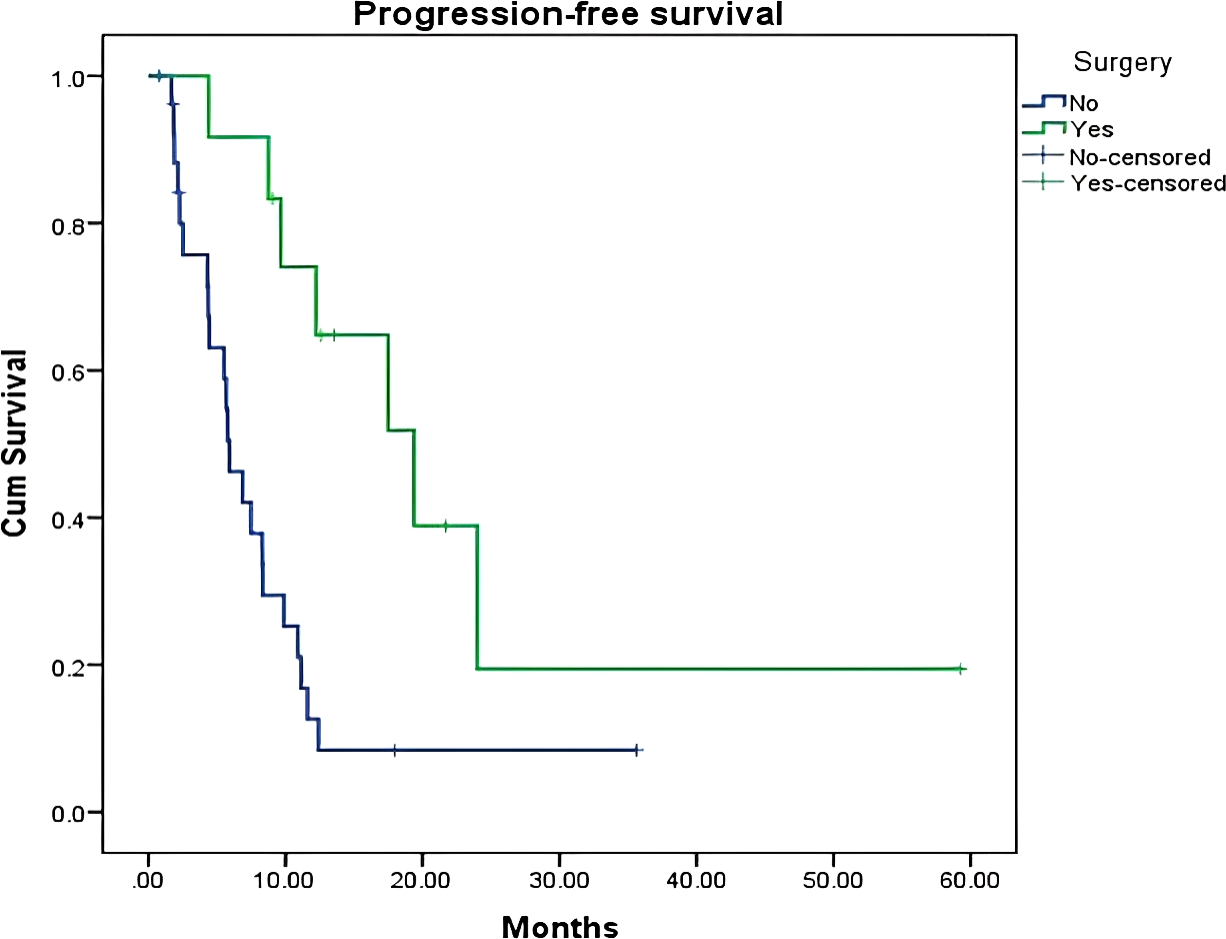
Kaplan–Meier curves of progression−free survival comparing patients who underwent surgical resection with those who did not.

The median overall survival (OS) for the entire cohort was 30 months (95% CI: 17.75 - 42.56 months) (**Figure 1**). The median progression-free survival (PFS) was 8 months (95% CI: 5.65 - 11.89 months) (**Figure 2**). Patients who underwent surgery after NAT had a numerically longer median OS compared to those who did not undergo surgery (30 months vs. 25 months), but this difference was not statistically significant (p = 0.26). However, patients who underwent surgery had a significantly longer median PFS compared to those who did not undergo surgery (19.6 months vs. 5.8 months; p = 0.008) (**Figure 3**). Elevated pre-treatment CA19–9 levels were associated with a trend towards worse OS (HR 1.8, 95% CI 0.9–3.6), but this association did not reach statistical significance. Similarly, an elevated pre-treatment NLR (>3) was associated with a trend towards worse PFS (HR 1.5, 95% CI 0.7–3.1), but this association was also not statistically significant. The PLR (>225) was not significantly associated with either OS or PFS (**Table 3**).

**Table 3:** Univariate analysis of various variables, OS, and PFS.

Variables	n	OS			PFS			Logistic regression		
		Median survival time	95%.CI	P-value	Median survival time	95%.CI	P-value	Odd ratio	95%.CI	P-value
Whole group	39	30	(17.75, 42.56)	NA	8	(5.65,11.89)	NA			
ECOG PS				0.4			0.8			0.8
0-1	30	30.16	(20.81, 39.50)		9.6	(5.88,13.43)		1		
2	6	20.4	(10.27, 30.59)		8.7	(92.35,15.19)		1.16	.180 -7.557	
NLR				0.3			0.9			0.9
<=3	28	25.19			8.7	(6.65,10.89)		1		
>3	10	Not reached	–	–	5.7	(3.39,8.17)		1.07	.220-5.210	
PLR				0.7			0.8			0.9
<=255	31	30.16	(10.23,50.08)		8.3	(5.89,10.79)		1		
>225	7	25.19	(17.76, 32.6)		10.9			0.97	.159-5.998	
Weight loss				0.3			0.07			0.7
Yes (1,2)	21	Not reached	–		17.5	(5.80,29.14)		1		
No (3)	11	20.7	(16.44,25.01)		8.3	(4.16,12.45)		1.33	.267-6.653	
Gender				0.2			0.5			0.6
F		Not reached	–		9.6	(2.13,17.18)		1		
M		25.19	(14.79,35.59)		8.3	(5.36,11.32)		0.7	.173-2.836	

ECOG, Eastern Cooperative Oncology Group; OS, Overall Survival; PFS, Progression Free Survival; NLR, Neutrophil Lymphocyte Ratio; PLR, Platelet Lymphocyte Ratio.

Multivariate Cox regression analysis was performed to assess the independent prognostic significance of ECOG performance status, CA19–9 levels, NLR, and PLR on OS and PFS. The results of the multivariate analysis showed numerical differences in both OS and PFS for these variables, but none of these differences reached statistical significance, likely due to the limited sample size. Multi-agent regimens achieved numerically superior conversion (36-40% vs 20%, Fisher’s exact p=0.12) and PFS (11.8-12.4 vs 6.9 months, log-rank p=0.16),Post-NAT CA 19–9 normalization was achieved in 39% of patients and served as a robust predictor of surgical outcome. Normalization correlated with a significantly higher resection rate (71% vs. 18%; Fisher’s exact p=0.03) and a notable trend toward improved progression-free survival (PFS; 14.2 vs. 6.1 months; HR 0.52; p=0.12 (**Table 4**)). These dynamic metrics demonstrated superior prognostic utility over static baseline assessments.

**Table 4:** Prognostic value of dynamic CA 19–9 assessment.

CA19–9 dynamic metric	n	Resection rate	Median PFS (months)	HR	P-value
Post-NAT Normalization	7/18(39%)	5/7 (71%)	14.2	0.52 (0.22-1.25)	0.12
No Normalization	11/18(61%)	2/11 (18%)	6.1		
≥ 50% Decline	11/18(61%)	7/11 (64%)	13.8	0.61 (0.28-1.35)	0.18
>50% Decline/progression	7/18(39%)	0/7 (0%)	5.4		

## 4 Discussion

This study provides an evaluation of the impact of NAT on conversion rates, surgical outcomes, and survival in patients with unresectable or borderline resectable PDAC in Saudi Arabia. The cohort size (n=39; 2.6 patients/year across three tertiary centers) reflects epidemiological realities in Saudi Arabia, where PDAC age-standardized incidence increased modestly from 1.9 to 3.0/100,000 males and 1.2 to 1.8/100,000 females (2005-2020) (5, 6). This low accrual rate reflects real-world barriers to neoadjuvant therapy referral, including late presentation (>80% advanced disease at diagnosis), limited multidisciplinary infrastructure during the study period, and stringent tumor board selection favoring patients with ECOG 0–2 performance status (77% in cohort) and absence of metastases. While this may introduce selection bias toward fitter NAT candidates, the study provides novel, generalizable data from an understudied Middle Eastern population where pancreatic cancer outcomes lag global benchmarks.

The median age of 60 years (range: 29–79 years) aligns with broader demographic patterns, where PDAC incidence increases sharply after age 50, with peak rates observed in individuals over 65 (34, 35). A male predominance (64%) was noted, consistent with global sex disparities in PDAC incidence, which are attributed to differential exposure to risk factors such as smoking and occupational hazards (36).

The resection outcomes observed in our cohort align with contemporary evidence on PDAC. Our cohort demonstrated a 31% overall resection rate following NAT, with R0 margins achieved in 33% of resected cases, consistent with meta-analyses reporting 20–33% resection rates for initially unresectable PDAC (37, 38). These results, however, remain below the 64–85% R0 rates documented in borderline resectable pancreatic cancer (BRPC) cohorts receiving FOLFIRINOX-based regimens (39, 40) likely reflecting our population’s mixed composition of Borderline Resectable Pancreatic Cancer (BRPC) and locally advanced pancreatic cancer (LAPC) cases, where vascular involvement persists as a critical barrier to margin clearance (40, 41). The PREOPANC trials provide crucial context for these findings. The PREOPANC-1 trial established that neoadjuvant gemcitabine-based chemoradiotherapy improved 5-year overall survival to 20.5% versus 6.5% with upfront surgery, alongside higher R0 rates (63% vs. 31%) in resectable/BRPC patients (11, 42). Conversely, the PREOPANC-2 protocol, directly comparing FOLFIRINOX against gemcitabine-based regimens, aims to optimize neoadjuvant strategies through enhanced systemic therapy delivery (43). These trials underscore the paradigm shift toward NAT, which not only increases R0 likelihood by reducing tumor bulk and perineural invasion (44, 45) but also selects biologically favorable tumors less prone to occult metastases (37, 38). Notably, the Alliance A021501 trial achieved 65.3% R0 rates in BRPC patients using modified FOLFIRINOX ± hypofractionated radiotherapy, surpassing historical benchmarks and demonstrating the potential of intensive protocols (39, 40). This aligns with meta-analyses showing NAT improves median survival (19.8 vs. 13.3 months) and doubles lymph node negativity rates (30.9% vs. 15.0%) compared to upfront surgery (37, 46). In LAPC specifically, FOLFIRINOX achieves 20–33% R0 rates after conversion therapy, while total neoadjuvant therapy (TNT) combining chemotherapy and radiotherapy elevates rates to 30–40% (44, 47).

The Conko-007 trial further corroborates this, reporting 35% R0 rates with neoadjuvant chemotherapy plus chemoradiation despite lacking survival benefits in interim analyses (40, 47). Margin-negative resection remains the strongest survival determinant, with 5-year rates of 24.2% after R0 versus 4.3% post-R1/R2 resections (48). Our cohort’s 33% R0 achievement assumes particular significance given the established correlation between margin clearance and long-term outcomes (41). Thirteen patients (33%) in our cohort received FOLFORINOX as NAT, 10 patients (26%) received Gemcitabine and Capecitabine,10 patients (26%) received single-agent gemcitabine, and 6 patients (15%) received other regimens. On the other hand 6 patients (15%) received neoadjuvant radiation.

Emerging data suggest optimal outcomes require multimodal approaches. The Dutch PREOPANC-1 trial demonstrated superior locoregional control with chemoradiotherapy (11, 42), while Alliance A021501 highlights FOLFIRINOX’s systemic efficacy (39). Meta-analyses of nine randomized trials (n=1,194) confirm NAT’s survival advantage (HR 0.73) over upfront surgery, particularly when combining chemotherapy and radiotherapy (37, 40). However, debates persist regarding radiation sequencing-PREOPANC-2’s comparison of FOLFIRINOX versus gemcitabine-based protocols may clarify optimal regimens (49). As treatment intensification protocols evolve, personalized approaches balancing efficacy and toxicity will be essential to maximize survival gains in this challenging malignancy (37, 40).

Subgroup analysis revealed superior conversion rates among multi-agent neoadjuvant regimens—FOLFIRINOX 5/13 (38%) and GEMCAP 4/10 (40%)—compared to gemcitabine monotherapy 2/10 (20%), aligning with meta-analyses reporting 20-33% resection rates for initially unresectable PDAC following NAT (10). This regimen heterogeneity reflects temporal evolution in clinical practice: gemcitabine-based therapies predominated pre-2011, while FOLFIRINOX adoption accelerated post-PRODIGE-4 publication, demonstrating survival superiority (11.1 vs 6.8 months). Multi-agent regimens trended toward better outcomes (conversion 36-40% vs 20%, Fisher’s exact p=0.12; PFS 11.8-12.4 vs 6.9 months, log-rank p=0.16), consistent with PREOPANC trial findings favoring intensive regimens. These observations validate modern intensification strategies within historical cohort constraints (11, 14, 15). The prognostic trends observed in our cohort align with established literature while highlighting important nuances in biomarker utility for PDAC. Our finding of elevated pre-treatment CA19–9 levels (HR 1.8, 95% CI 0.9–3.6) demonstrating a non-significant trend toward worse OS resonates with meta-analyses reporting pooled HRs of 1.72 (95% CI 1.31–2.26) for CA19-9 >305 kU/L in resected PDAC (50). Post-NAT CA19–9 normalization (39%; PFS HR 0.52, 95% CI 0.22-1.25) demonstrated superior prognostic discrimination compared to baseline levels, aligning with established evidence that treatment-induced normalization or significant decline predicts improved survival across PDAC stages (16–18). Specifically, post-therapeutic CA19–9 normalization identifies biologically favorable disease more reliably than pretreatment elevation (our cohort baseline HR 1.8 OS), supporting dynamic biomarker monitoring to guide NAT continuation, surgical timing, and patient selection in conversion therapy protocols. Our observed NLR >3 trend (HR 1.5, 95% CI 0.7–3.1 for PFS) mirrors meta-analyses demonstrating NLR’s prognostic value (HR 2.21, 95% CI 1.45–3.36) (51), though recent NAT studies show conflicting results regarding NLR dynamics and survival (52, 53). The PLR >225 lacked prognostic significance in our analysis, contrasting with meta-analyses reporting pooled HRs of 1.24–1.28 for elevated PLR (54, 55), potentially reflecting differences in cutoff selection or the modulating effects of NAT on platelet biology (56).

The potential survival benefit of adjuvant chemotherapy following conversion surgery warrants emphasis. In our cohort, most resected patients with available records (8/9, 89%) received gemcitabine-based adjuvant therapy, a strategy associated with improved disease-free survival during the treatment era. Contemporary analyses increasingly suggest that intensified postoperative regimens may further enhance outcomes for patients who undergo conversion after modern neoadjuvant approaches such as FOLFIRINOX. These observations support the possibility of therapeutic synergy between neoadjuvant treatment intensification (e.g., FOLFIRINOX, nab-paclitaxel) and optimized adjuvant systemic therapy (57). Prospective studies incorporating standardized adjuvant protocols after conversion surgery are needed to define the optimal postoperative strategy.

Several limitations must be acknowledged. The retrospective design and relatively small sample size limit the statistical power and generalizability of the findings. Heterogeneity in NAT regimens, surgical techniques, and postoperative care may introduce confounding variables. Additionally, the lack of standardized criteria for response assessment and resectability, as well as incomplete data on some biomarkers and outcomes, may affect the robustness of the conclusions. A notable limitation of this multicenter retrospective analysis is the incomplete documentation of adjuvant therapy for 25% of patients who underwent resection. Such heterogeneity in postoperative care could confound the attribution of long-term Overall Survival (OS) specifically to the neoadjuvant-to-surgery sequence. However, the primary endpoint of the PFS, measured from the commencement of NAT to disease progression or death, remains a robust and reliable surrogate for neoadjuvant efficacy. The significant PFS benefit observed in the surgical cohort (HR 0.42, p = 0.008) underscores the efficacy of the neoadjuvant approach in achieving disease control, largely independent of subsequent postoperative interventions.

Despite these limitations, this study provides important regional data and reinforces key principles in the management of advanced PDAC. Future research should focus on prospective, multicenter studies to validate these findings and refine patient selection criteria.

In conclusion, NAT facilitates resection in approximately one-third of patients with initially unresectable PDAC, with a significant PFS benefit in patients undergoing subsequent surgical resection.

## Data Availability

The raw data supporting the conclusions of this article will be made available by the authors, without undue reservation.
